# Experience in endoscope choice for neuroendoscopic lavage for intraventricular hemorrhage of prematurity: a systematic review

**DOI:** 10.1007/s00381-024-06408-6

**Published:** 2024-05-27

**Authors:** Catherine E. Wassef, Ulrich W. Thomale, Melissa A. LoPresti, Michael G. DeCuypere, Jeffrey S. Raskin, Shreya Mukherjee, Kristian Aquilina, Sandi K. Lam

**Affiliations:** 1 Department of Neurosurgery, University of Rochester Medical Center, Rochester, NY USA; 2 Pediatric Neurosurgery, Charité Universitätsmedizin Berlin, Berlin, Germany; 3 Division of Pediatric Neurosurgery, Lurie Children’s Hospital, 225 E Chicago Ave, Box 28, Chicago, IL 60611 USA; 4 Department of Neurosurgery, Northwestern University Feinberg School of Medicine, Chicago, IL USA; 5 Department of Pediatric Neurosurgery, Great Ormond Street Hospital, London, UK

**Keywords:** Intraventricular hemorrhage, Prematurity, Hydrocephalus, Preemie IVH, Neuroendoscope, Neuroendoscopic lavage

## Abstract

**Objective:**

Intraventricular hemorrhage (IVH) of prematurity occurs in 20–38% of infants born < 28 weeks gestational age and 15% of infants born in 28–32 weeks gestational age. Treatment has evolved from conservative management and CSF diversion of temporizing and shunting procedures to include strategies aimed at primarily clearing intraventricular blood products. Neuroendoscopic lavage (NEL) aims to decrease the intraventricular blood burden under the same anesthetic as temporizing CSF diversion measures in cases of hydrocephalus from IVH of prematurity. Given the variety of neuroendoscopes, we sought to review the literature and practical considerations to help guide neuroendoscope selection when planning NEL.

**Methods:**

We conducted a systematic review of the literature on neuroendoscopic lavage in IVH of prematurity to examine data on the choice of neuroendoscope and outcomes regarding shunt rate. We then collected manufacturer data on neuroendoscopic devices, including inflow and outflow mechanisms, working channel specifications, and tools compatible with the working channel. We paired this information with the advantages and disadvantages reported in the literature and observations from the experiences of pediatric neurosurgeons from several institutions to provide a pragmatic evaluation of international clinical experience with each neuroendoscope in NEL.

**Results:**

Eight studies were identified; four neuroendoscopes have been used for NEL as reported in the literature. These include the Karl Storz Flexible Neuroendoscope, LOTTA^®^ system, GAAB system, and Aesculap MINOP^®^ system. The LOTTA^®^ and MINOP^®^ systems were similar in setup and instrument options. Positive neuroendoscope features for NEL include increased degrees of visualization, better visualization with the evolution of light and camera sources, the ability to sterilize with autoclave processes, balanced inflow and outflow mechanisms via separate channels, and a working channel. Neuroendoscope disadvantages for NEL may include special sterilization requirements, large outer diameter, and limitations in working channels.

**Conclusions:**

A neuroendoscope integrating continuous irrigation, characterized by measured inflow and outflow via separate channels and multiple associated instruments, appears to be the most commonly used technology in the literature. As neuroendoscopes evolve, maximizing clear visualization, adequate inflow, measured outflow, and large enough working channels for paired instrumentation while minimizing the footprint of the outer diameter will be most advantageous when applied for NEL in premature infants.

## Introduction 

Intraventricular hemorrhage (IVH) is a common and severe neurological complication of prematurity, occurring in approximately 20% of very-low-birth-weight preterm neonates (born at < 32 weeks of gestational age) [1]. Pathophysiologically, IVH arises from the germinal matrix, which is located underneath the ventricular ependyma near the head of the caudate nucleus and from which neuronal and glial cell precursors arise [2, 3]. The overall incidence of IVH worldwide is between 20 and 40% of all infants weighing below 1500 g, with increased incidence in lower birth weight and gestational age [4, 5]. In the United States, about 12,000 premature infants develop IVH each year [2]. Despite improvements in survival rates, with some reports as high as 70%, IVH can cause posthemorrhagic hydrocephalus, cerebral palsy, and intellectual disabilities [4, 6]. The long-term impact of IVH can be devastating for patients and their families; thus, it is imperative to develop an understanding of this condition to assist in advancing treatment strategies.

The mainstay of treatment of posthemorrhagic hydrocephalus (PHH) in large enough infants (> 2 kg) is the ventriculoperitoneal shunts (VPSs) [7]. However, there is no standard consensus treatment for hydrocephalus following IVH in low-weight (< 2 kg) infants. The management of PHH in this group has evolved over time. Initially, medical therapies aimed at correcting coagulopathy; later therapies targeted mediators of perinatal instability including respiratory distress with the use of corticosteroids [4]. Surgical treatment centered on cerebrospinal fluid diversion, initially through serial lumbar punctures, ventricular aspiration by transfontanelle taps, placement of ventricular access devices, and ventriculosubgaleal shunts, with progression to ventriculoperitoneal shunting when silicone shunts were popularized in the 1950s [2, 4, 8]. More recent surgical interventions have been aimed at reducing intraventricular clot burden. The drainage, irrigation, and fibrinolytic therapy (DRIFT) trial used bilateral EVDs to instill a fibrinogen activator and irrigate the ventricles for several days, thus diluting the inflammatory milieu thought to cause secondary injury after IVH and relieving intraventricular pressure [9]. DRIFT observed procedural complications including secondary IVH (35% of treatment cohort) and cerebrospinal fluid (CSF) infection without difference in mortality or need for subsequent CSF shunt surgery [10]. The 2-year and 10-year follow-up data showed important outcomes: those who received DRIFT treatment had reduced mortality and lower rates of severe disability compared to standard lumbar puncture treatment at 2-year follow-up. In addition, those treated with DRIFT had better cognitive outcomes at 2-year and at 10-year follow-up compared to standard treatment. There is growing evidence for the rationale to remove blood products of IVH of prematurity and eventually reduce shunt dependency and improve outcomes [4, 9, 11–14].

While temporizing measures are often needed in this neonate population due to the risk of complications related to VP shunt insertion in preterm infants under 2 kg, such as infection, valve blockage by debris or clot, recurrent hemorrhage, and peritoneal malabsorption [15–17], neuroendoscopic lavage (NEL) in conjunction with temporizing treatment (VAD/VSGS) has emerged as new techniques and technologies develop. NEL aims to intervene on the source of compromised CSF flow dynamics and ultimately improve odds for shunt avoidance and better neurodevelopment [14, 18]. It is performed in the controlled environment of the operating room for a finite amount of time under sterile conditions, as opposed to continuous lavage in the intensive care unit with DRIFT. NEL also has the advantage of direct visualization, whereas DRIFT was performed through one frontal and one contralateral occipital ventricular catheter. DRIFT also lacked the advantage of improving CSF communication through septostomy or endoscopic third ventriculostomy which is possible with NEL in which the ventricles are directly inspected for adequate hematoma evacuation, hemostasis, and CSF flow patency.

Given the evolution of neuroendoscopy, the variety of techniques applied in the management of PHH, and the clinical burden of IVH of prematurity, we aimed to conduct a systematic review of the literature regarding IVH of prematurity to identify the types of neuroendoscopes used in NEL. A recent consensus approach to NEL indicated that both rigid and flexible endoscopes are appropriate options for this procedure [19]. We pair this with an assessment of the manufacturer information regarding widely available neuroendoscopes presently on the market to examine and evaluate the neuroendoscopes used in NEL with the hopes of informing endoscope selection and experience when used in the treatment of IVH of prematurity.

## Methods

### Search strategy

A systematic review following the Preferred Reporting Items for Systematic Reviews and Meta-analyses (PRISMA) guidelines [20] was conducted to identify all neuroendoscopes used in the literature for NEL in PHH of prematurity. PubMed MEDLINE, Embase, Cochrane Library, and EBSCO host CINHAL databases were searched from their inception through July 2, 2023. Search terms included “neuroendoscopic lavage,” “neonatal,” and “intraventricular hemorrhage.” Additionally, original studies reported in meta-analyses were examined to determine if they met inclusion criteria.

### Selection criteria

Duplicates were removed, and non-full-text English language journal articles including abstracts, conference presentations, and editorials were also excluded. All remaining articles were screened based on title and abstract. After title and abstract exclusion, the remaining articles underwent full-text review. Articles were selected based on the inclusion and exclusion criteria. Articles were included if they met the following criteria: studied patients who were premature infants with intraventricular hemorrhage who underwent NEL and identified the neuroendoscope(s) used in NEL. Articles were excluded if studying a different patient population than premature infants with IVH, did not assess the intervention of NEL, and did not detail the endoscope used.

### Data extraction

Data extracted from all included studies were study design, number of infants included in each study who underwent NEL, neuroendoscope used, and clinical outcomes. Primary outcome measured included VPS rate after NEL; the secondary outcome measured included the impact of NEL on CSF profile, namely, red blood cell (RBC) count and protein. A summary of included studies and outcomes of interest are detailed in Table 1.

**Table 1:** Studies reporting NEL shunting rates and endoscope usage

**Included study**	**Year**	**Study design**	**N**	**NEL procedures**	**Endoscope(s) used**	**Key findings**	**Study quality**
Sartori et al. [21]	2021	Case report	1	1	Karl Storz flexible endoscope	‐ Feasibility study ‐ Transfontanelle ultrasound as an adjunct ‐ 0% VPS Rate	Low
Schulz et al. [14]	2014	Retrospective cohort, matched	29	19	MINOP	‐ 58% VPS rate with NEL vs. 100% with conventional treatment	Moderate
Tirado-Caballero et al. [22]	2020	Retrospective cohort	46	83	MINOP	‐ Statistically significant decrease in CSF protein and blood products before and after lavage ‐ 58.7% VPS rate ‐ 1-year shunt failure rate 50%	Moderate
Etus et al. [23]	2018	Multicenter retrospective cohort, matched	74	23	GAAB	‐ 60.8% VPS rate with NEL vs. 93.1% with conventional treatment ‐ 77.2% VPS rate after VSGS	Moderate
Behrens et al. [18]	2020	Retrospective cohort	42	42	MINOP or LOTTA	‐ 59% VPS rate with NEL	Low-moderate
d'Arcangues et al. [24]	2018	Retrospective cohort	56	65	MINOP or LOTTA	‐ 57% VPS rate with NEL	Moderate
Honeyman et al. [25]	2022	Retrospective cohort	26	28	MINOP	‐ 65.4% VPS rate with NEL ‐ Both CSF protein and RBC count were significantly reduced with NEL	Moderate
Schaumann et al. [26]	2021	Retrospective cohort	116 (92 with IVH)	104	MINOP or LOTTA	‐ 58.8% VPS rate with NEL	Moderate

*N* number, *NEL* neuroendoscopic lavage, *IVH* intraventricular hemorrhage, *VPS* ventriculoperitoneal shunt, *VSGS* ventriculosubgaleal shunt, *RBC* red blood cells, *CSF* cerebrospinal fluid

### Quality assessment

The risk of bias was evaluated according to the Cochrane ROBINS-I guidelines [27]. The quality score for each included study is indicated in Table 1.

### Statistical analysis

Given the heterogeneity of data, no meta-analysis was performed.

### Review of neuroendoscope manufacturer data

After the identification of all scopes used for NEL, manufacturer data was obtained for each scope. Manufacturer data from Karl Storz and Aesculap was reviewed for device specifications for all included and popularly-used neuroendoscopes. Scopes reviewed included Karl Storz Neuro Flexible Endoscope, Little LOTTA^®^, LOTTA^®^, and GAAB as well as the Aesculap MINOP^®^ and PaediScope. The MINOP^®^ InVent scope was not included in this review: it has a notably larger size with an outer diameter of 8.3 mm. The InVent has not been reported in neuroendoscopic lavage to date, which may be in part due to the large endoscope in relation to the small size of the neonatal population and typically thin cortical mantle of the brain.

Data collected included flexibility of the scope, product ID, outer diameter of the scope, working channel diameter, scope length, inflow and outflow mechanisms, viewing angle/degrees of visualization, irrigation mechanisms (manual or automatic), sterilization requirements, and instruments available for use in the working channel (Tables 2 and 3). We paired this with subjective advantages and disadvantages clinically observed in the practice of multiple pediatric neurosurgeons across international centers. This data was tabulated to provide a summative consideration guide for users selecting neuroendoscopes for NEL in pediatric populations.

**Table 2:** Neuroendoscope specifications

**Name**	**Product ID**	**Outer diameter (mm)**	**Working channel diameter**	**Inflow (mm)**	**Outflow (mm)**	**Length (cm)**	**Degrees of visualization**	**Automated irrigation**	**Advantages**	**Disadvantages**
Storz Neuro Flexible Endoscope	11161VK	2.9	1.2	N/A	N/A	35	< 270	No	Many degrees of visualization, only flexible option, small diameter, digital camera source improves visualization	Chemical sterilization, one channel, no inflow/outflow option, fiberoptic camera source results in an optical screen effect, leaming curve with handling the scope
Storz Little Lotta	28164LLA	3.6	1.6	0.8	0.8	18	0, 6	No	Autoclavable, irrigation adaptor, fiberoptic light transmission incorporated, stabilizing introducing sheath	Small outflow channel increases resistance
Storz Lotta	28008AA	6.1	2.9	1.6	1.6	26	0	No	Autoclavable, bimanual dissection, balanced inflow/outflow, can irrigate and aspirate through working portal, stabilizing introducing sheath	Large outer diameter
	28164LA	6.1	2.9	1.6	1.6	18	6	No		
	28164LAB	6.1	2.9	1.6	1.6	18	30	No		
Storz pediatric GAAB Set	28161AMA	4.5	1.3	1	1	21	0	No	Autoclavable, small outer diameter	Crescent-shaped, not easy to irrigate, limited availability
Aesculap MINOP Intraventricular endoscopic system	FF399R	6	2.2	1.4	1.4	15	0, 30	Yes	Autoclavable, balanced inflow/outflow, can irrigate and aspirate through working portal, can fit Artemis Penumbra, paired introducing sheath	Large outer diameter
	FF398R	4.6	N/A	0.8	0.8	15	0, 30	Yes	Autoclavable, balanced inflow/outflow, can irrigate and aspirate through working portal, paired with introducing sheath	No specific working channel, small irrigation channel
Aesculap PaediScope	PF010A	3	1.2	0.8	0.8	15	0	Yes	Autoclavable, balanced inflow/outflow, can irrigate and aspirate through working portal, paired with introducing sheath	Small irrigation channel

**Table 3:** List of recommended instruments and holding systems by endoscope manufacturer

**Scope**	**Compatible instruments**	**Product ID**		**Diameter × length**
Karl Storz Neuro Flexible Endoscope	KS Cup Biopsy Grasper	11003KA		1 mm × 60 cm
	KS Grasping Forceps	11003KB		1 mm × 60 cm
	Disposable Cook Cup Biopsy Forceps	G15054		3.3 ft × 115 cm
Karl Storz Little LOTTA	Scissors, single-action jaws	28161SC		1.3 mm × 30 cm
	Biopsy Forceps, double-action jaws	28161SB		1.3 mm × 30 cm
	Grasping Forceps, double-action jaws	28161SG		1.3 mm × 30 cm
	Bipolar Coagulation Electrode	28161SF-SB		1.3 mm × 30 cm
	Forceps, for ventriculostomy	28160TV		1 mm × 30 cm
Karl Storz Large LOTTA	CLICKLINE Biopsy Forceps, single-action jaws	28164LF		2.7 mm × 30 cm
	CLICKLINE Scissors	28164LB		2 mm × 30 cm
	CLICKLINE Biopsy Forceps, double action	28164LC		2 mm × 30 cm
	CLICKLINE Ventriculostomy Forceps	28164LD		2 mm × 30 cm
	CLICKLINE Grasping Forceps	28164LE		2 mm × 30 cm
	Scissors, pointed, lightly curved jaws, double action	28162EM		1.7 mm × 30 cm
	Scissors, pointed, single action	28162FP		1.3 mm × 30 cm
	Forceps, for ventriculostomy	28160TV		1 mm × 30 cm
	Biopsy Forceps, flexible, double-action jaws	28160ZJ		1 mm × 30 cm
	BDV bipolar	28164BD		2.4 mm × 30 cm
	TAKE-APART^®^ Bipolar Forceps	28164BDVT		2.4 mm × 30 cm
	Bipolar Forceps Insert	28164FGV-S		Single-use
	Guillotine Knife	28164LG		2.7 mm × 30 cm
	Bipolar Coagulation Electrode	28762KB		1.7 mm × 30 cm
Karl Storz GAAB	Injection Needle	28162PK		1.7 mm
	Deflecting Mechanism	28161LD		2.9 mm × 38 cm
	Grasping Forceps	28162U		2.7 mm × 30 cm
	Biopsy Forceps, single-action jaws	28162ZE		2.7 mm × 30 cm
	Scissors, pointed, single-action jaws	28162EP		2.7 mm × 30 cm
	Scissors, pointed, slightly curved jaws, double-action	28162EM		1.7 mm × 30 cm
	Biopsy Forceps, double-action jaws	28162Z		1.7 mm × 30 cm
	Forceps, for ventriculostomy	28160TVX		1.7 mm × 30 cm
	Coagulating Electrode, bipolar	28762KB		1.7 mm
	Coagulating Electrode, unipolar	28762K		1.7 mm
MINOP (Aesculap)	Artemis Penumbra aspiration device			2.1 mm
	MINOP Micro Scissors, sharp/blunt	FF385R/FF386R		2.0 mm × 2.65 cm
	MINOP Grasping and Dissecting Forceps	FF388R		2.0 mm × 2.65 cm
	MINOP Biopsy Forceps	FF387R		2.0 mm × 2.65 cm
	MINOP Surgical Micro Forceps, 1X2 teeth	FF389R		2.0 mm × 2.65 cm
	MINOP Monopolar Blunt Electrode	GK361R		1.1 mm
	MINOP Monopolar 90° Hook Electrode	GK362R		2.2 mm
	MINOP Monopolar Needle Electrode	GK363R		1.1 mm
	MINOP Monopolar 45° Hook Electrode	GK364R		2.2 mm
	MINOP Monopolar 70° Hook Electrode	GK365R		2.2 mm
	MINOP Monopolar J Hook Electrode	GK366R		
	Bipolar Fork Electrode	GK360R		2.1 mm
	Suction Cannula, blunt tip 0°	FH606SU		
	Suction Cannula, sharp tip 45°	FH607SU		
MINOP (Aesculap) PaediScope	Micro scissors	FF373R		1.0 mm × 2.5 cm
	Micro grasping and dissecting forceps	FF374R		1.0 mm × 2.5 cm
	Micro biopsy forceps	FF378R		1.0 mm × 2.5 cm
	Blunt electrode	GK361R		1.1 mm × 2.55 cm
	Needle electrode	GK363R		1.1 mm × 2.55 cm

**Introducers/sheaths/holding systems**				
**Endoscope system**	**Device**		**Product ID**	**Outer diameter**
Storz Little LOTTA	Operating sheath		28164LLS	4.5 mm (13.5 Fr) × 13.3 cm
Storz LOTTA	Operating sheath		28164LS	6.8 mm × 13 cm
Storz GAAB	Operating sheath		21862 BS	6.5 mm
Storz Neuro Flexible Endoscope	Peel-away sheath			11 Fr
Aesculap MINOP	Introducer (peel-away)		FH604SU	7.3 mm (22 Fr)
Storz GAAB	Holding system		28272 KKA	N/A
Storz LOTTA or Little LOTTA	Holding system		28272 HB, 28272UKN, 28172HR	N/A
Aesculap MINOP	Holding arm and universal holder		FF168R, RT046P	N/A

## Results

### Systematic review findings

**Fig. 1:**
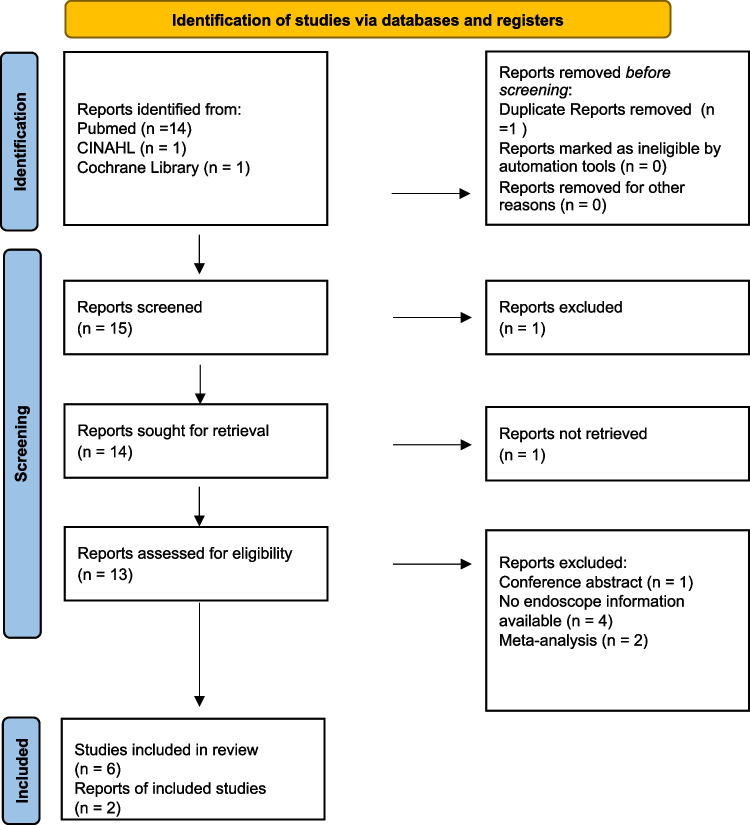
PRISMA flow diagram for included studies

Of 26 resultant articles, eight met our inclusion criteria (Fig. 1). Seven articles were retrospective cohort studies; one was a case report (Table 1).

A total of 365 NEL procedures were performed. Four neuroendoscopes were identified as used in premature infants, some of which had varying endoscopic camera options for a range of viewing angles. Neuroendoscopes used in NEL reports included the following: Minimally Invasive Operating Procedure (MINOP^®^, Aesculap, Tuttlingen, Germany), intraventricular neuroendoscopic system LOTTA^®^ (Karl Storz, Tuttlingen, Germany), Storz GAAB neuroendoscope, and Storz flexible neuroendoscope (Karl Storz, Tuttlingen, Germany) [28–30]. Three studies reported the use of the MINOP or LOTTA. Three studies reported the use of the MINOP only, and one study each reported the use of the GAAB and flexible neuroendoscopes. The Little LOTTA^®^ (Karl Storz, Tuttlingen, Germany) has been used for other pediatric neuroendoscopic procedures, but no reports were found in the NEL literature.

Primary outcome measured was the VPS rate after NEL. This ranged from 0% in the one case report to 77.2%; however, among the seven included retrospective studies, the VPS rate was 57–77.2% [14, 18, 21–26].

### Endoscope specifications, advantages, and disadvantages

**Fig. 2:**
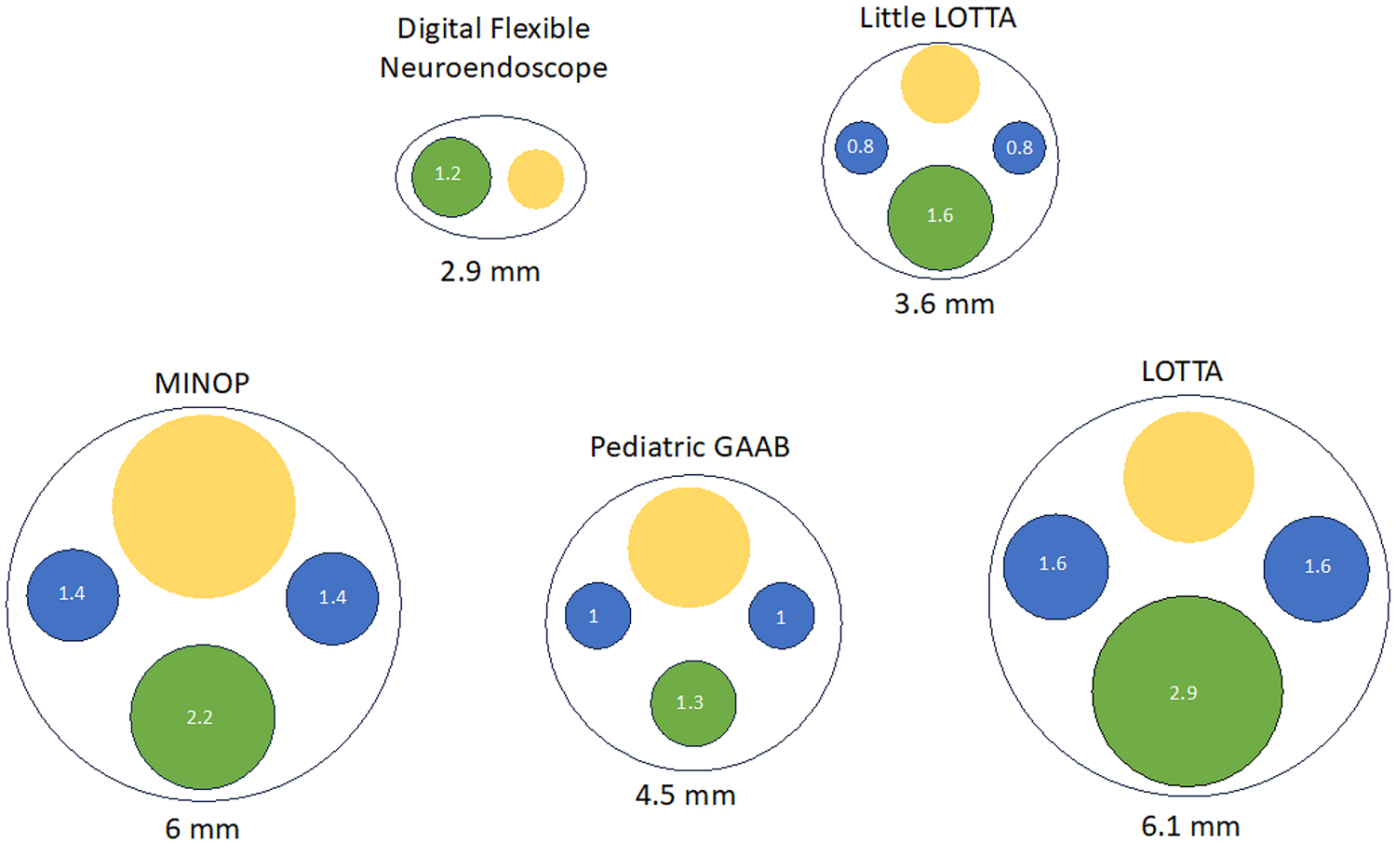
Cross-section of references endocscopes. Displayed is the scaled cross-sectional view of each endoscope tip with diameters of each channel displayed within the circle (in millimeters). Blue = inflow/outflow, green = working channel, yellow = optics lens

Specifications of each neuroendoscope used in NEL as well as those which could potentially be reviewed for intraventricular work are included in Table 2. We detail the flexibility, outer diameter of the scope, working channel configuration and diameter, inflow/outflow mechanism, scope length, visualization degrees, and irrigation automation. Each endoscope can be stabilized with a holding arm. Figure 2 provides a comparative scaled depiction of the cross-sectional view of all five endoscopes. Advantages and disadvantages are listed for comparison (Table 2) to provide a summative display when weighing options for neuroendoscope selection.

#### Aesculap MINOP^***®***^

The most reported neuroendoscope for NEL is the Aesculap MINOP^®^ intraventricular neuroendoscopic system with a convex tip [14, 18, 22, 24–26], which contains two irrigation/outflow channels, one working channel, and an optical endoscope channel. Advantages include an automated irrigation option and a larger working channel (2.2 mm), which can accommodate instruments including biopsy forceps, dissecting forceps, scissors, and various monopolar and bipolar electrodes (Table 3). The optic endoscope channel offers exchangeable optics (0 and 30° view) for individual purposes of intervention. The working channel can accommodate other equipment such as some ultrasonic aspiration devices for hematoma aspiration [31], typically considered for use in adult patients. Bimanual work may be feasible if one of the inflow/outflow channels is used as a second working channel with smaller instruments. Disadvantages include a large outer diameter (6 mm) which creates a larger tract in the cortical mantle, which may portend a higher risk of CSF leak in pediatric patients with thin cortical mantles and large ventricles. There are also smaller MINOP trocars with a 4.6 mm outer diameter, with no working channel, two smaller irrigation/outflow channels (0.8 mm), and an optic channel, and 3.2 mm outer diameter trocar with an optic channel only. MINOP also offers a peel-away sheath for stabilization of the endoscope’s tract given the dynamic nature of endoscopic surgery.

#### Storz LOTTA^***®***^

The second most reported neuroendoscope is the Storz LOTTA^®^ for intraventricular neuroendoscopy (LOTTA) with a flat slightly angled tip [18, 24, 26], which may be selected in different prefixed versions of two endoscopic camera angles: 6 and 30° [29]. The advantage of this scope is the largest working channel (2.9 mm) allowing the use of larger instruments, as well as a balanced inflow and outflow given the larger diameter of both channels (1.4 mm). Bimanual work is feasible if one of the inflow/outflow channels is used as a second working channel with smaller instruments, though as these are larger than the MINOP working and inflow/outflow channels, these may afford larger instruments in comparison. The LOTTA is aided by an operating sheath with an outer diameter of 6.8 mm that may be affixed to a stabilizing arm and can hold the operating scope, which may be large when used in neonates. The outer diameter of this scope is 6.1 mm, and it may appear large in pediatric patients with thin cortical mantles and large ventricles and may theoretically pose a risk of CSF leak.

#### Storz little LOTTA^***®***^

The Storz Little LOTTA^®^ intraventricular neuroendoscope has a smaller outer diameter (3.6 mm) with a flat slightly angled tip and is available with a 6° viewing angle [29]. Little LOTTA^®^ also has the added benefit of an operating sheath (outer diameter 4.6 mm). Although it provides a working channel (1.6 mm) and inflow/outflow channels (0.8 mm), there can be some resistance in the outflow causing an imbalance in the inflow and outflow of irrigation. Little LOTTA^®^ has not been reported for IVH evacuation in the literature but has been used for endoscopic fenestrations and third ventriculostomy in neonates [26]. Anecdotally based on individual authors’ experience, the volume turnover through inflow and outflow channels is not enough to achieve CSF clearance in a reasonable time effort. Thus, little LOTTA has not been selected routinely for use in NEL procedures by the authors at their respective institutions.

#### Storz pediatric GAAB^***®***^

The Storz pediatric GAAB set has been reported for NEL [23], but it does not appear to be widely used in other case series. The pediatric GAAB working channel is 1.3 mm, the inflow and outflow channels are 1 mm each, and the outer diameter is 4.5 mm; making it one of the smaller neuroendoscopes [32]. The regular GAAB has a 6.5 mm outer diameter, which has not been reported to have been used in IVH of prematurity. The disadvantage is that it has not gained widespread use, and Etus et al. [23] reported difficulty with irrigation due to the crescent shape of the endoscope.

#### Storz neuro flexible endoscope^***®***^

The Storz flexible neuroendoscope is the only flexible endoscope reported for intraventricular hemorrhage evacuation in the literature [21]. Its flexible nature allows for up to 270° visualization, and it boasts a small outer diameter of 2.9 mm [30]. Flexible neuroendoscopy has gone through an evolution, with two camera/light options, fiberoptic and digital, each affording different optics based on institutional resources and selection. The digital neuroendoscope will afford enhanced optics for future developments as the fiberoptic neuroendoscope has an optical screen effect which may cloud the image. There is one working channel without additional inflow or outflow channels, thus fluid volume and flow regulation must be closely monitored when used for lavage. However, it may be used with a peel-away sheath to create an “external” outflow around the endoscope.

## Discussion

We conducted a systematic review identifying the neuroendoscope selection when planning NEL in a neonate with PHH following IVH of prematurity. We identified four different neuroendoscopes used in NEL in eight included studies and provided a summative experience from the literature and our authors’ experiential data regarding the use of five widely available neuroendoscopes. We found that the MINOP^®^, LOTTA^®^, and little LOTTA^®^ systems have many similarities, including sterilization requirements, holding arm, instruments, and inflow/outflow channels. While these are significant benefits, scopes with smaller outflow channels often lead to challenges with irrigation regulation, and larger scopes have larger footprints. The flexible neuroendoscope is unique due to its wide degree of visualization; however, there is an associated learning curve with its use, the sterilization process is more complex, and it is limited by no regulated inflow/outflow mechanism. While understanding the pros and cons of each endoscope option for NEL is instrumental to success, each scope has its role and benefit, and thus, each should be considered in the context of the surgery planned for the individual patient.

### Considerations to select the appropriate neuroendoscope

**Fig. 3:**
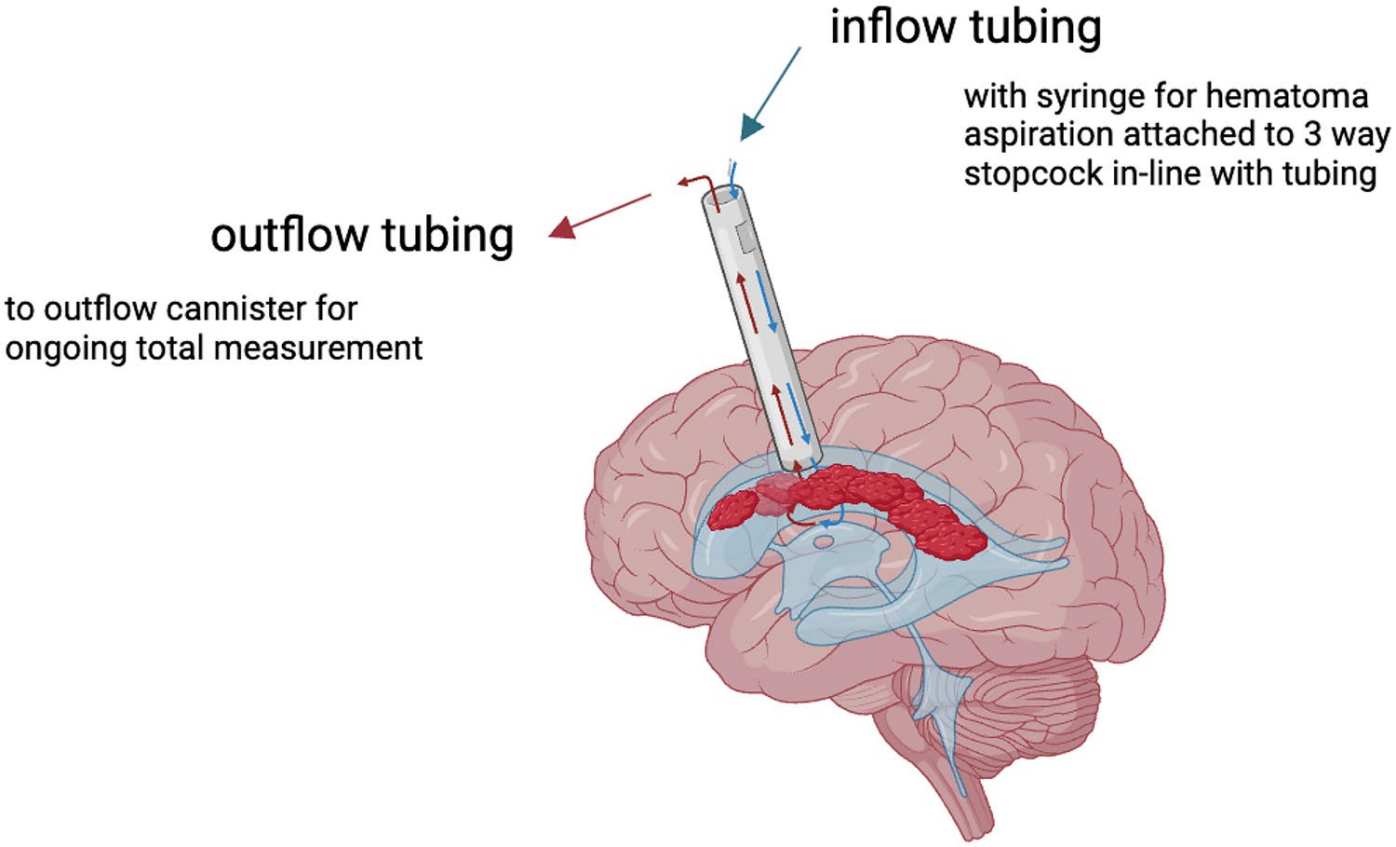
Conceptual diagram of neuroendoscopic lavage. Inflow and outflow for irrigation is key to the procedure

While surgical technique, user experience, and practice patterns/preferences may impact which endoscope is used, several factors can be considered in endoscope selection. First, the patient, procedure, and goal of surgery must be examined. In patients with large ventriculomegaly relative to the cortical mantle, efforts should be made to minimize the risk of CSF leak and wound healing complications by additional meticulous measures for multilayer wound closure. Reports of CSF leak after NEL range from zero to 25% (after repeated NEL procedures) [14, 22, 25]; as such, the authors utilize a conical rolled piece of dry Gelfoam^®^ to occlude and seal the endoscope tract upon removal. The diameter of the endoscope and thus the corticotomy plays a crucial role in the efficiency of the procedure which correlates with the inner diameter of the inflow and outflow channels. The same concept applies to the size of the endoscope in relation to the foramen of Monro. Because NEL may involve irrigation and evacuation of the third ventricle, the endoscope needs to be small enough to navigate through the foramen of Monro. Another aspect is the total length and weight of the camera of each endoscope, which causes different handling strategies during the slight movements navigating throughout the ventricle, especially in the small head of a neonate. See Fig. 3 for a conceptual diagram of the NEL procedure.

In patients with thick clot burden, larger working channels which may accommodate different instruments [31] or with automated inflow/outflow mechanisms may provide a benefit in terms of function and operative time, often a critical consideration in this young, small, vulnerable population. However, the larger footprint may leave a larger area of access-related tissue injury and CSF pathway. Although a sizeable neuroendoscope diameter may appear large compared to a neonate’s head, the effectiveness of the procedure determines the success of evacuation of the intracranial blood products, and thereby, reducing further brain damage by way of the inflammatory cascade and CSF dynamics may outweigh the risks of the endoscope’s size [14, 15, 18, 22–26, 33, 34].

Environmental and institutional factors must also be considered, such as the availability of scopes at the institution; the availability of chemical sterilization processes may limit the ability to clean and house a scope, and support staff and supplies to help set up, troubleshoot, and maintain/service equipment as needed may limit use. This is of particular importance when considering the flexible neuroendoscope as it requires special chemical sterilization infrastructure and regulatory permission, limiting its use in areas where this method of sterilization is not available. For instance, it is not currently available for use in Europe due to regulatory guidelines.

For NEL, flexible neuroendoscopes may appear to be an attractive endoscope choice with a smaller diameter and more maneuverability. Its flexibility allows access to the tip of the endoscope to a wider area within the contralateral ventricle and the posterior third ventricle without torsion at the cortical entry point. This may facilitate direct aspiration of hematoma in these regions. However, we have personally noted some potential disadvantages: the flexible neuroendoscopes contain one working channel with no additional inflow and outflow channels. There is no ability to quantify the inflow and outflow of fluid for ventricular irrigation. There can be a danger of overfilling of the ventricular system with irrigation and the development of high intracranial pressure without adequate outflow. If a peel-away sheath is used for introducing the neuroendoscope into the ventricle, there is unmeasured egress of CSF around the neuroendoscope throughout the procedure, which may incur some risk of saline on the neonate’s body causing hypothermia during the intervention.

### Additional NEL considerations in surgical planning

While the selection of a neuroendoscope is critical to the execution of a successful lavage, additional factors are important to consider when planning surgery, for example, the mode of irrigation, automated or manual, as well as the type and temperature of the irrigation. Given the small size of the patient, balance of electrolytes and fluids is critical. The temperature of the irrigation to be used through the ventricular system is also important. Given that neonates have small body weight and other comorbidities, it is important to monitor all aspects of the operating room environment and temperature control. In order to avoid hypothermia, there are multiple safety measures we recommend: (1) the irrigation should be kept at body temperature in a sterile fluid warming system, (2) the patient should be draped carefully to make sure the body is sealed from the cranial operative field to keep the body dry and warm, and (3) the multidisciplinary operating room team should be cognizant of such risks of fluctuations in body temperature during surgery so as to keep active all ambient room temperature and warming measures.

### Lessons learned

While there are some factors which cannot be accommodated, some learning curves and modifications can assist with technique development. For instance, with the use of the flexible endoscope, the holding arm can provide bimanual dexterity for scope manipulation to enhance navigation throughout the ventricular system to the prepontine cistern and beyond. Additionally, increased experience with the flexible scope can lead to enhanced navigation through the ventricular system. The surgical team is an important area of focus. Involvement of two surgeons in any NEL procedure is invaluable with any endoscopic equipment. This dynamic procedure requires not only navigation of the endoscope, but also coordinated use of the working channel with close team communication assisting with irrigation, suction, passing instruments through the scope, and troubleshooting. In other circumstances, modifications of equipment can be used to enhance the experience. For example, a recent study [35] detailed a method of automated irrigation using the irrigating bipolar machine, serving as an adjunct in scopes without an inflow/outflow mechanism: the bipolar irrigation tubing fits within the Luer-lock irrigating port of some neuroendoscopes such as the Aesculap MINOP and PaediScope and Storz LOTTA and little LOTTA systems. This concept can be expanded to any Luer-lock irrigating port, making automated irrigation available to all scopes with inflow and outflow channels.

### Limitations

There are several limitations to our study. First, only published studies with available full-text manuscripts were included, which portends publication bias and an overestimation of the number of positive and significant study results. Furthermore, only studies written in or translated into the English language were included, potentially excluding successful interventions and studies from other areas of the world. Additionally, the overall quality of evidence reviewed was moderate since most studies were retrospective cohorts. As there was no standardized management guideline, each institution adapts its own protocol to NEL, in terms of when to intervene, how to intervene, and when to shunt patients with PHH, precluding statistical analysis for comparison across studies. Additionally, we acknowledge the limited level of evidence provided from experiential data and manufacturer information. Given the level of expertise required to perform neuroendoscopy, we believe that this experiential evidence is key to understanding nuanced advantages and disadvantages when selecting a neuroendoscope, areas for improvement, and considerations when planning for NEL. Accrued experience can be transmitted for improved outcomes and optimal usage of technology to perform new and advanced procedures, such as NEL.

## Conclusions

We conducted a systematic review identifying several neuroendoscopes used in NEL for IVH of prematurity. We paired this with manufacturer information and experiential considerations to provide a summative assessment and inform endoscopic selection for use in NEL. We believe that key considerations include irrigation with balanced inflow and outflow, working channel size and instrumentation pairings, and quality and degrees of visualization. As neuroendoscope technology evolves, a smaller outer diameter endoscope with adequate inflow/outflow diameters and working channel, with a wide range of view and a variety of associated instruments to pass through the working channel, would be most advantageous in the neonate pediatric population for NEL.
